# Limited Antineutrophil Cytoplasmic Antibodies (ANCA)-Negative Granulomatosis With Polyangiitis: Successful Response to Rituximab

**DOI:** 10.7759/cureus.41826

**Published:** 2023-07-13

**Authors:** Maria Margarida Andrade, Manuel Fernandes, Sara Freire, Diogo Cruz

**Affiliations:** 1 Internal Medicine, Hospital de Cascais, Dr. José de Almeida, Cascais, PRT; 2 Pulmonology, Hospital de Cascais, Dr. José de Almeida, Cascais, PRT; 3 Clínica Universitária de Medicina I, Faculdade de Medicina da Universidade de Lisboa, Lisboa, PRT

**Keywords:** anca-negative, immunosuppressors, vasculitis, rituximab, granulomatosis with polyangiitis

## Abstract

Granulomatosis with polyangiitis (GPA), a systemic vasculitis, is commonly characterized by the presence of antineutrophil cytoplasmic antibodies (ANCA). However, a subset of patients with limited disease may exhibit ANCA negativity. In this article, we report the case of a 40-year-old female diagnosed with GPA with intolerance to methotrexate titration and glucocorticoid therapy, leading to the initiation of rituximab treatment. Subsequently, the patient exhibited sustained clinical, laboratory, and radiological improvement. The identification of limited GPA has important therapeutic implications as the effectiveness of the medical treatment in ANCA-negative GPA may differ. Rituximab has emerged as an optimal treatment, irrespective of ANCA status, offering prolonged responses and a favorable tolerance profile in these patients.

## Introduction

Granulomatosis with polyangiitis (GPA) is a systemic vasculitis primarily linked to the presence of antineutrophil cytoplasmic antibodies (ANCA) [1]. However, ANCA are not found in a subset of patients with GPA. ANCA negativity is associated with limited disease affecting the lungs, throat, nose, and ears without renal involvement [1,2]. Rituximab, a monoclonal anti-CD20 antibody, has shown efficacy in treating autoimmune rheumatic diseases, including systemic GPA [2,3]. In this article, the authors present a case of a patient diagnosed with limited ANCA-negative GPA with a favorable response to rituximab. This case underscores the effectiveness of B-cell depletion therapy even without detectable autoantibodies.

## Case presentation

A 40-year-old Caucasian woman, smoker, with well-controlled type 1 diabetes, was admitted to the Emergency Department due to exertional dyspnea, productive cough, nasal congestion, pleuritic chest pain, and chronic arthralgias of both hands. On physical examination, there were no signs of arthritis, and bibasal crackles were noted on pulmonary auscultation. Blood tests revealed anemia, neutrophilic leukocytosis with no eosinophilia, a C-reactive protein (CRP) of 15 mg/dL, and an erythrocyte sedimentation rate (ESR) of 77 mm/h. Kidney and liver function tests were normal. Urinalysis did not present proteinuria or hematuria.

**Figure 1:**
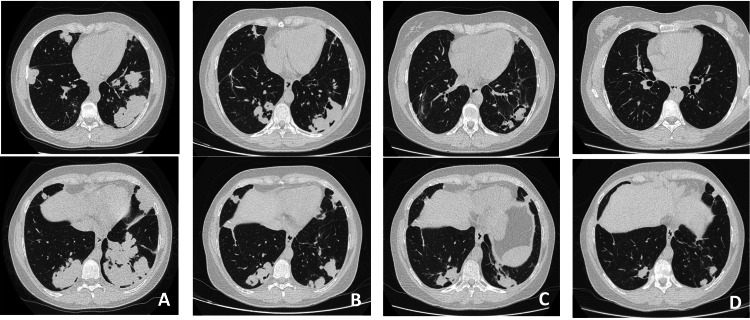
Chest computed tomography evolution Evolution of the imaging pattern on chest computed tomography - at admission (A), one month after starting immunosuppression with methotrexate and steroids (B), two months and 18 months after taking rituximab (C, D, respectively).

Chest computed tomography (CT) exhibited multiple bilateral solid parenchymal nodular lesions, predominantly located in the lower lobes, with the largest lesion measuring 7.5 cm in diameter. These nodules were confluent, displayed pleural contact, and had no air bronchogram (Figure 1A).

Initially, the patient started antibiotics for a presumed diagnosis of pneumonia. However, due to the lack of improvement, a CT-guided transthoracic needle biopsy (TNB) was performed. The biopsy results revealed thickened vessel walls and a mixed, solid, necrotizing lung lesion consistent with a sclerosing granuloma. Subsequent 2-deoxy-2-[18F]fluoro-D-glucose (FDG)-positron emission tomography (PET) scan showed extensive lung lesions with an atypical pattern (Standardized Uptake Values (SUV) 3.9-5.4) along with uptake observed in the left ethmoid sinus (SUV 4.8), thus raising the suspicion of GPA. The patient underwent a flexible bronchoscopy with bronchoalveolar lavage (BAL). The BAL cytology revealed a high concentration of macrophages without neoplastic cells. For further pathological characterization and GPA diagnosis confirmation, a video-assisted thoracoscopy surgery (VATS) biopsy was performed. The pathological evaluation of the lung tissue obtained confirmed the presence of necrotizing granulomatous inflammation with concurrent vasculitis, thus establishing the diagnosis of GPA. ANCA were negative. Steroids (prednisone 1 mg/kg) and methotrexate (2.5 mg/week with subsequent titration) were started.

On subsequent chest CT reevaluation, there was a global radiological improvement with a reduction of the consolidations' dimensions bilaterally and a resolution of some (Figure 1B). Pulmonary function tests (PFTs) revealed a mild restrictive ventilatory pattern (forced vital capacity (FVC) of 69%, forced expiration in the first second (FEV1) of 75,5%, FEV1/FVC ratio of 79, total lung capacity of 79,6%) and a moderate decrease in carbon monoxide diffusing capacity (DLCO). In the six-minute walk test (6MWT), the patient only covered 292 meters, which makes up 55% of the predicted distance.

Due to oral ulcers that made oral feeding difficult, further titration of methotrexate was impossible beyond 12.5 mg/week. A switch to rituximab was then decided and there was clinical and laboratory improvement (CRP of 0.3 mg/dL and ESR of 17 mm/h). Two months after therapy with rituximab, the patient remained clinically stable, maintaining notorious imaging improvement (Figure 1C). In what concerns PFTs, the FVC improved to 81.5%. The 6MWT also improved to a distance that covered 85% of the predicted, without pauses. Six months after therapy with rituximab, the patient remains stable with no respiratory complaints. The chest CT imaging 18 months after the initial diagnosis is shown in Figure 1D. At two years follow-up, the patient maintains clinical and radiological remission.

## Discussion

GPA is a chronic small and medium vessel vasculitis, involving the airways, kidneys, and skin, with the formation of perivascular and/or extravascular granulomas. The presence of ANCA, particularly proteinase subtype 3, is commonly associated with GPA [1,2]. ANCA has been observed to induce neutrophil activation, resulting in a respiratory burst and the release of inflammatory cytokines [2]. However, approximately 10% of patients with GPA do not exhibit detectable ANCA levels [1,3].

In 1966, Carrington and Liebow described a subgroup of 16 patients with a limited form of GPA. These individuals presented necrotizing granulomatous lesions characteristic of GPA but had minimal vasculitic involvement and no evidence of glomerulonephritis [4]. Subsequent studies have recognized that some patients present with a nonsevere or limited form of GPA, which does not immediately endanger vital organs or life [2]. ANCA-negative cases are more commonly associated with nonsevere or limited disease, and this manifestation occurs frequently in women [5]. Patients with limited GPA typically have a longer disease duration and a higher likelihood of disease recurrence compared to those with systemic disease [6].

The diagnosis of GPA is based on clinical and laboratory findings [1]. Following the modified criteria established by the American College of Rheumatology, our patient met one clinical criterion (nasal congestion) and three other criteria (pulmonary nodules, granuloma on biopsy, and inflammation of nasal sinuses on imaging), totaling 8 points [7]. According to Stone's classification, the patient's disease can be categorized as limited since it meets the criteria but does not present immediate threats to critical organs or the patient's life [4,8].

ANCA-negative GPA is still not fully understood. Recent studies suggest that these patients constitute 40% of those with limited GPA affecting the upper airways and lungs [4]. Monitoring ANCA titers during follow-up may often reveal a decrease, but negative and persistently positive titers have been observed, with no clinical outcomes association [3]. Some evidence suggests that the clinical response is independent of ANCA status. Despite being ANCA-negative during their disease course, some patients achieve complete remission. Others become ANCA-negative without experiencing any clinical improvement [3].

Recognizing the limited form of GPA is crucial as the effectiveness of medical treatment in ANCA-negative cases may differ. While some cases exhibit a positive response to immunosuppressors, others may show poor responses to these agents [1]. Historically, cytotoxic agents like cyclophosphamide in combination with glucocorticoids have been the primary treatment approach for the past 50 years. However, this approach is associated with long-term complications such as infertility, myeloproliferative disorders, and transitional cell carcinoma of the bladder. Therefore, alternative regimens with reduced toxicity have been avidly sought [8]. Since the 1990s, it has been recognized that patients with limited GPA can be effectively treated with glucocorticoids and methotrexate [9]. The 2022 EULAR recommendations for the treatment of GPA propose a combination of steroids and rituximab for induction of remission in non-organ-threatening or non-life-threatening GPA (level of evidence 1B). Methotrexate or mycophenolate mofetil can be considered alternatives to rituximab [10]. This recommendation is based on the RAVE trial, which studied the use of rituximab vs. cyclophosphamide in a population mainly with severe ANCA-positive disease [11]. There are no randomized controlled trials in patients with non-organ-threatening GPA, especially in the ones that are ANCA-negative [10].

Rituximab, a chimeric monoclonal antibody targeting CD20 B-cell antigen, has been used to treat severe GPA [2]. The precise role of B-cells in GPA is not yet fully understood. B-cells acting as antigen-presenting cells to T-cells or providing additional co-stimulatory signals to them might be an explanation [2]. Another possibility is that self-reactive B-cells derived from distinct B-cell subsets undergo an alternative maturation process, maintaining CD20 expression during antibody production [2]. The addition of rituximab to the ongoing treatment of patients with refractory or relapsing GPA, as seen in our patient, results in a remarkable regression of constitutional and vasculitis-related symptoms, irrespective of ANCA status. These responses are sustained for several months. The treatment is generally well-tolerated [3].

## Conclusions

The presented case emphasizes the importance of recognizing the limited form of ANCA-negative GPA and the evolving treatment approaches in recent years. Rituximab has demonstrated significant efficacy in achieving remission of symptoms in GPA, regardless of ANCA status, through profound depletion of CD20-expressing cells. Further research is necessary to determine the optimal role of rituximab compared to traditional therapies like cyclophosphamide.
